# Functional study of *ZmHDZ4* in maize (*Zea mays*) seedlings under drought stress

**DOI:** 10.1186/s12870-024-05951-3

**Published:** 2024-12-19

**Authors:** Xiaowen Xie, Zhenzhen Ren, Huihui Su, Salah Fatouh Abou-Elwafa, Jing Shao, Lixia Ku, Lin Jia, Zhiqiang Tian, Li Wei

**Affiliations:** 1 College of Agronomy, Henan Agricultural University, Zhengzhou, Henan 450046 China; 2 Agronomy Department, Faculty of Agriculture, Assiut University, Assiut, Egypt

**Keywords:** *Zea mays*, HD-ZIP, Water deficit, Expression

## Abstract

**Background:**

Maize is a major feed and industrial crop and pivotal for ensuring global food security. In light of global warming and climate change, improving maize tolerance to water deficit is crucial. Identification and functional analysis of drought tolerance genes have potential practical importance in understanding the molecular mechanisms of drought stress.

**Results:**

Here, we identified a maize Homeodomain-Leucine Zipper I, *ZmHDZ4*, in maize seedlings that is associated with drought tolerance. We demonstrated that ZmHDZ4 has transcriptional activation activity, exclusively localized in the nucleus. Several *Cis*-acting elements associated with abiotic stress have been identified in the core promoter region of *ZmHDZ4*. Under drought-stressed conditions, transgenic maize plants overexpressing *ZmHDZ4* exhibited significantly higher relative water content and peroxidase (POD) and superoxidase dismutase (SOD) activities compared to wide-type plants, while displaying lower malondialdehyde (MAD) content. The expressions of *ZmMFS1-88*, *ZmGPM573*, and *ZmPHD9* were significantly repressed in the *ZmHDZ4*-OE plants under drought-stressed conditions, indicating that *ZmMFS1-88*, *ZmGPM573*, and *ZmPHD9* were the candidate target genes of *ZmHDZ4*.

**Conclusions:**

*ZmHDZ4* is involved in the regulation of drought stress tolerance in maize by participating in osmotic regulation, sugar metabolism pathways, and hormone regulation.

**Supplementary Information:**

The online version contains supplementary material available at 10.1186/s12870-024-05951-3.

## Background

Maize is a major feed and industrial crop, vital for ensuring global food security. In China, a major corn-producing country, there is a heightened focus on increasing yields and improving quality. However, the escalating impacts of global climate change are exacerbating water scarcity issues, with gradually decreasing precipitation and increasing evaporation, leading to more frequent and severe droughts [14, 27]. Drought is one of the foremost challenges facing the world today. As one of the 13 water-scarce countries in the world, China’s vast land area underscores urgent need to protect water resources, improve agricultural production, and strengthen drought prevention. Therefore, it is important to promote water-saving techniques in irrigation and improve water use efficiency. Moreover, leveraging bioinformatics and molecular breeding technologies to explore and improve maize’s inherent drought resistance capabilities and develop more drought-resistant varieties is paramount [28].

There are significant differences in water requirements across different growth stages of maize [19]. During the planting period, maize seeds must imbibe water equivalent to half of their weight for successful germination. In the seedling stage, the soil moisture content should be maintained between 60 and 70% of the field water capacity. As the crop progresses from the tillering to the tasseling stage, its water demand significantly increases. This demand peaks during the tasseling stage to the grain-filling stage. Even at the maturing stage, after the grain filling process is completed, maize requires a substantial amount of water resources. However, water demand gradually decreases after the milk mature stage. The impact of drought stress on maize plant morphology varies at different growth stages. During the seed germination period, drought stress can delay germination and result in stunted and weak plants. The seedling and early seedling stages are critical periods because maize exhibits heightened sensitivity to drought stress during these periods. Studies indicate that moderate drought stress during the seedling stage can promote root development, but prolonged drought stress can significantly reduce seedling establishment in the field, affecting their growth rate, photosynthesis, nutrient growth time, and reproductive growth time, ultimately leading to a yield reduction [8].

Drought resistance refers to a plant’s ability to perceive external water shortages and initiate corresponding drought resistance mechanisms to withstand external stress. These mechanisms primarily include avoidance, tolerance, and resistance to drought. Plants improve drought tolerance by changing their life cycles, regulating their physiological processes, altering their morphology, or activating the expression of drought-responsive genes [39]. As drought severity and duration escalate, reliance solely on avoidance mechanisms becomes untenable for sustaining normal plant growth. Consequently, drought tolerance assumes a pivotal role, regulating the antioxidant defense systems, transcription factors, osmotic stress substances, and signaling molecules [13].

Transcription factors are a class of proteins that can bind to DNA molecules and regulate the expression of target genes by activating or inhibiting transcription, which have been reported to play important roles under drought stress, such as *AP2/ERF*, *MYB*, *bZIP*, *HD-Zip*, *NAC*, and *WRKY* [6, 16, 24, 34, 39]. The sequence of homeodomain-leucine zipper (HD-Zip) proteins encompasses 60 highly conserved amino acid homeodomain (HD) regions and adjacent leucine zipper (LZ) regions. Based on the homology of their structural domains [2]. HD-Zip transcription factors have been identified across various plants, including Arabidopsis, rice, wheat, and maize [7, 17, 20, 33]. The HD-Zip family can be divided into four different subfamilies, HD-Zip I to HD-Zip IV, with HD-Zip I, containing only the HD and LZ domains, serving as key regulatory factors in plant development and stress response. The expression of these family members is predominantly influenced by drought, salt, low temperature, and osmotic stress [29]. Lots of studies have demonstrated the significance of HD-Zip transcription factors in conferring drought tolerance in various plant species. For instance, the overexpression of *ATHB6* increases the drought resistance of transgenic maize plants by activating the expression of reactive oxygen species-related genes [9]. Additionally, *NaHD20* can regulate abscisic acid (ABA) accumulation in tobacco and activate the expression of drought-related genes under drought stress [22]. Similarly, overexpression of *TaHDZipI-5* in wheat increases drought tolerance and frost resistance [32], while the overexpression of *ZmHDZ10* in maize positive regulates drought and salt tolerance in Arabidopsis [36]. Notably, Qiu et al. [20] systemically analysed the HD-Zip transcription factors in maize based on the transcriptome data under drought-rewatering treatment and found that *ZmHDZ4*, -6, -9, -14, -27, -32, and − 40 are key regulatory genes through co-expression network analysis, with *ZmHDZ4* demonstrating a positive response to drought stress [20]. In this study, *ZmHDZ4*-overexpressing transgenic maize lines were developed using *Agrobacterium*-mediated transformation, and the function of *ZmHDZ4* was verified through the evaluation of phenotypic, physiological, and biochemical indicators. Using DAP-seq and EMSA, the binding motifs of *ZmHDZ4* in the genome were analysed and validated. The downstream target genes regulated by *ZmHDZ4* were selected and verified using the dual-luciferase reporter system and real time-quantitative polymerase chain reaction (RT-qPCR) analysis, elucidating the regulatory network of *ZmHDZ4* under drought stress. These findings lay a solid theoretical foundation for studying drought tolerance mechanism in maize.

## Methods

### Plant materials and stress treatments

The maize inbred line Yu882, provided by Professor Lixia Ku, was utilized in this study. Yu882 seeds were grown in a nutrient soil: vermiculite (3:1) mixture in the growth chamber. The light intensity was maintained at 300 µmol m^− 2^⋅s^− 1^ with a light/dark cycle of 16/8 h. The temperature in the chamber was kept constant at 26 ± 2℃, and the relative humidity was maintained at 70%. For assessing responses to high salinity and drought stresses, v3-stage-seedlings were transferred to Hoagland nutrient solution contains either 20% PEG 6000 (to simulate drought conditions) or 200 mmol L^− 1^ NaCl (to induce high salinity stress), respectively. The solution was changed every 48 h. To evaluate responses to high temperature (HT, 37℃) stress, v3-stage-seedlings were exposed to a day/night high temperature of 37℃ in the growth chamber. Fully expanded leaves were collected every 24 h after treatment, till 96 h. Samples were immediately frozen in liquid nitrogen and then stored at − 80℃ for further analyses. Samples from three individuals were combined to create one biological replicate, with three biological replicates for each treatment.

The full-length coding sequence (CDS) of *ZmHDZ4* was amplified and cloned into the plant transformation vector pFGC5941 under the control of the *cauliflower mosaic virus 35 S* (*CaMV35S*) promoter. The freeze-thaw approach was employed to introduce the vector into the *Agrobacterium tumefaciens* LBA4404 strain. The *ZmHDZ4*-overexpression (*ZmHDZ4-*OE) transgenic plants were generated through *Agrobacterium*-mediated transformation, utilizing the immature embryos from maize inbred line B104 as a receptor, B104 referred to as the wild-type (WT). Maize genetic transformations were conducted by Beijing Bomeixingao Technology Company, which also provided the seeds of the WT and *ZmHDZ4*-OE plants.

*ZmHDZ4*-OE and WT plants were grown in pots containing a 3:1 soil: vermiculite mixture in the greenhouse. Three-leaf-old seedlings from both *ZmHDZ4*-OE and WT plants were exposed to natural drought stress by withholding irrigation for 15 days. Meanwhile, the control treatment (well-watered) was normally irrigated. Leaves from the *ZmHDZ4*-OE and WT plants were sampled for RT-qPCR and physiological parameters analyses. The drought stress experiment was carried out with three biological replicates.

Relative water content (RWC), and the activities of malondialdehyde (MDA) content, superoxide dismutase (SOD) and peroxidase (POD) were measured following the procedures described by Ren et al. [24].

### RNA extraction and RT-qPCR analysis

Trizol reagent (TaKaRa, Dalian, China) was employed to extract the total RNA from the sampled leaves according to the manufacturer’s protocol. An aliquot of the extracted RNA was employed to synthesize the first-strand cDNA (10 mL) using the Prime-script™ RT reagent Kit with gDNA Eraser (TaKaRa, Dalian, China) according to the manufacturer’s instructions. RT-qPCR assay was performed using SYBR^®^*Premix Ex Taq II* (2×) (TaKaRa, Dalian, China) as provided in the manufacturer’s protocol, using the CFX96 Connect Real-Time System (Bio-Rad, Hercules, CA, USA). The relative gene expression was calculated using the 2^−ΔΔCT^ method [15]. The *18 S* RNA was chosen as an internal control. RT-qPCR assays were carried out using three biological and three technical replicates. The specific primer sequences are given in Supplementary Table S1.

### Subcellular localization and transcriptional activation assay of ZmHDZ4

The full-length coding sequence of *ZmHDZ4* without the stop codon was cloned using specific primers containing the *Kpn*I and *Sal*I restriction sites (Supplementary Table S1). The sequence was then inserted into the pMDC83-GFP vector. The fused plasmid of pMDC83-GFP-*ZmHDZ4* and the negative control (pMDC83-GFP empty vector) were separately transformed into the *Agrobacterium tumefaciens* EHA105 strain which was then injected into the leaves of *Nicotiana benthamiana* plants. After agroinfiltration, the plants were placed in a greenhouse at 25℃ for 48 h under dark conditions. Green fluorescent protein (GFP) fluorescence signals were then detected using a Zeiss LSM 700 confocal laser scanning microscope (Germany) at 488 nm.

The yeast strain AH109 (Weidi, Shanghai) containing *HIS3* and *lacZ* reporter genes was employed to estimate the transcriptional activity of *ZmHDZ4*. The full-length coding sequence of *ZmHDZ4* was cloned into the pBD-GAL4 vector via *Bam*HI/*EcoR*I to generate the pBD-GAL4-ZmHDZ4 recombinant vector. The pBD-GAL4-*ZmHDZ4*, pGBKT7 (negative control, empty vector) and pGAL4 (positive control, a full GAL4 transcription factor, encompassing both the transcriptional activation domain and the DNA-binding domain) were transformed into the yeast strain AH109. The yeast cells were initially cultured on a selective medium SD/-Trp. Subsequently, well-growing colonies were selected and transferred to selective plates SD/-Trp/-His/-Ade and SD/-Trp/-His/-Ade/+X-gal. The plates were subsequently placed in an incubator for 3–5 days at 30℃, after which the transcriptional activation was evaluated.

### Electrophoretic mobility shift assay (EMSA)

The electrophoretic mobility shift assay (EMSA) was carried out by amplifying the full-length cDNA of *ZmHDZ4* using primers harboring the *Bam*HI and *Not*I restriction sites (Supplementary Table S1), followed by restriction digestion of the PCR amplicons with corresponding restriction enzymes, and finally by inserting them into the pGEX-4T-1 vector digested with the same restriction enzymes. The fusion proteins of GST and GST-ZmHDZ4 were expressed in the *Escherichia coli* BL21 and purified by GST-tag Protein Purification Kit (Beyotime, P2262) according to the manufacturer’s protocol. Oligonucleotide probes (Supplementary Table S1) were synthesized and labeled according to the Invitrogen Technology standard procedure. The standard 20 µl reaction mixture utilized for EMSA that contains 20 ng of the purified ZmHDZ4 fusion protein, 5 ng of DIG-labeled annealed oligonucleotides, 2 µl of 10× binding buffer (100 mM Tris, 500 mM KCl and 10 mM ithiothreitol, pH 7.5), 1 µl of 50% (v/v) glycerol, 1 µl of 100 mM MgCl2, 1 µl of 1% (v/v) Nonidet P-40, 1 µl of 1 mg ml^− 1^ poly(dI–dC) and double-distilled water was used. The reaction was incubated for 20 min at 25 ℃, electrophoresed in 6% (w/v) polyacrylamide gels, and then transferred to N^+^ nylon membranes (Millipore) in 0.53× TBE (Tris-Borate- EDTA) buffer for 30 min at 380 mA and 4 ℃. DIG-labeled DNA detection was performed using the LightShift™ Chemi-luminescent EMSA kit (Thermo Fisher Scientific). The bands were visualized using the Chemiluminescent Western Blot Detection Kit (Thermo Fisher Scientific).

### DNA affinity purification and sequencing (DAP-seq) experiment and data analysis

DAP-seq assay was implemented as previously described by Bartlett et al. [4]. NEB Next^®^ DNA Library Prep Master Mix set for Illumina kit (NEB #E6040S) was used to construct the DAP-Seq genomic DNA (gDNA) library. In brief, about 10 ug DNA was fragmented, followed by end-repaired, addition of A-tailing, and ligation of an adapter. The *Sgf*I and *Pme*I restriction enzymes were implemented to clone the full-length coding sequence of *ZmHDZ4* into the pFN19K HaloTag^®^ T7 SP6 Flexi^®^ vector to generate Halotag-ZmHDZ4 fusion protein. The purified fusion protein and gDNA library were co-incubated, followed by washing and elution. The eluent (DAP-seq library) was sequenced on the Illumina platform with a 150 bp paired-end strategy.

To generate clean data, the fastp software was used to remove low-quality reads from the raw data. The Bowtie2 (v2.3.4.3) software was used to map clean reads to the maize B73 reference genome (Zm-B73-REFERENCE-GRAMENE-4.0). The MACS (v2.2.7.1) software was used to identify *ZmHDZ4*-binding sites. Genes exhibiting peaks located within 3 kb upstream or downstream were defined as target genes.

### Dual luciferase (Dual-luc) assay

The dual-luc assay was performed as described by Su et al. [25]. Briefly, about ~ 2000 bp of the promoter regions from the potential target genes were amplified from the B73 maize genotype and cloned into the pGreenII-0800-LUC vector to generate reporter genes. The full-length coding sequence of *ZmHDZ4* was cloned into the pCAMBIA-1300 vector, generating the effector. The dual-luc assays in *N. benthamiana* leaves were conducted and measured using Glo-Max^®^20/20 Luminometer (Cat# E5311, Promega). Three independent measurements were carried out for each gene.

### Statistical analysis

Statistical analysis was performed using the SAS software. All values reported in this study were the means of three independent replicates. The statistical significance of differences was determined according to Duncan’s method at *p < 0.05, **p < 0.01.

## Results

### Characteristics of ZmHDZ4

**Fig. 1:**
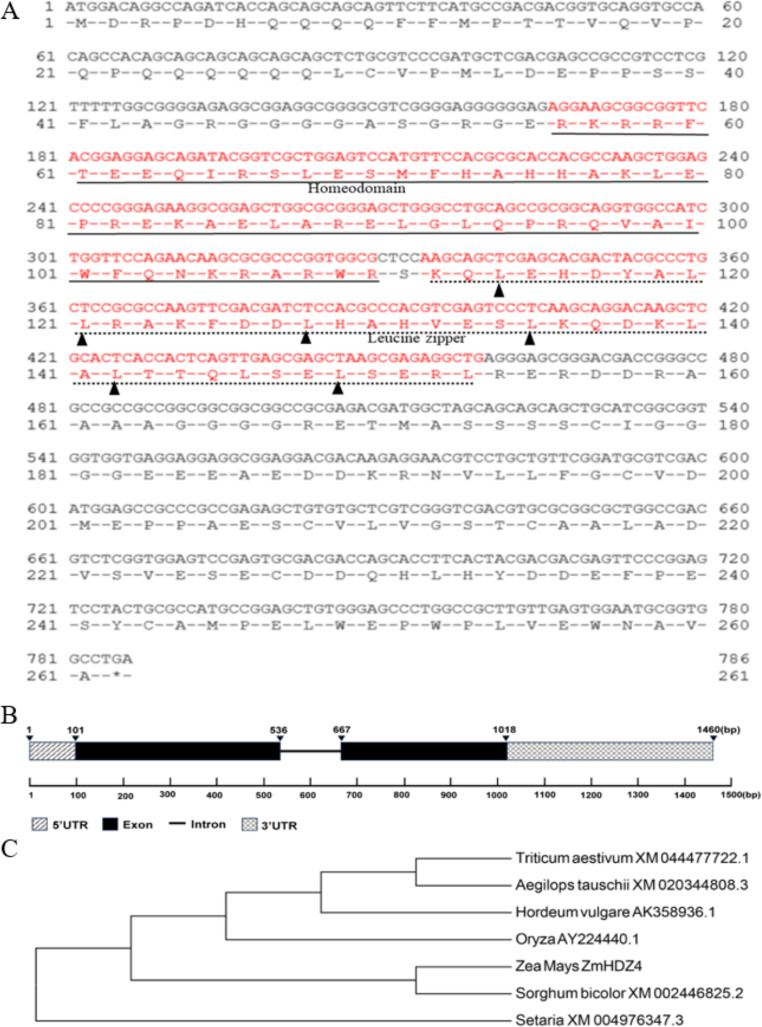
Characteristics analysis of *ZmHDZ4*. **A** ORF and encoded amino acid sequence of *ZmHDZ4* (red is the domain region). **B** A schematic diagram exhibiting the exon-intron structure of *ZmHDZ4*. **C** The phylogentic relationship of ZmHDZ4 with the homologous sequences in other species

Sequence alignment showed that the open reading frame of *ZmHDZ4* is 786 bp, comprising two exons and one intron and encoding 261 amino acids (Fig. 1A, B). The calculated molecular weight of the encoded protein is 29.38 kDa, with an isoelectric point value is 4.76. Structural domain analysis revealed that the protein encoded by *ZmHDZ4* contained two domains: the homeodomain (HD) and its neighboring leucine zipper (Zip)domain. Notably, the Zip domin contains a leucine residue every seven amino acids, repeated six times. The HD and Zip domains are located at 56–110 and 112–153 positions of the amino acid sequence, respectively, belonging to the HD-ZIP family. To further investigate the evolutionary relationship, the MEGA 6.0 software was used to construct a phylogenetic tree, and the result showed that ZmHDZ4 protein was most closely related to the HD-ZIP protein family in sorghum, consistent with the alignment results, with distant relationships observed with *Hordeum vulgare* AK358936.1 and *Aegilops tauschii* XM020344808.3 (Fig. 1C). Analysis of *Cis*-acting elements using the online PlantCARE software showed that the promoter region of *ZmHDZ4* contains multiple elements related to abiotic stress, such as the low-temperature stress-related element (LTR), CGTCA-motif, TGACG-motif, and TGA-element elements involved in jasmonic acid and auxin signal transduction, as well as ACGT-containing ABA response elements (ABRE) related to the ABA signal transduction pathway under drought stress. This suggests a potential role of *ZmHDZ4* in phytohormone signal transduction and abiotic stress response.

### Subcellular localization and transactivation activity analysis of ZmHDZ4

**Fig. 2:**
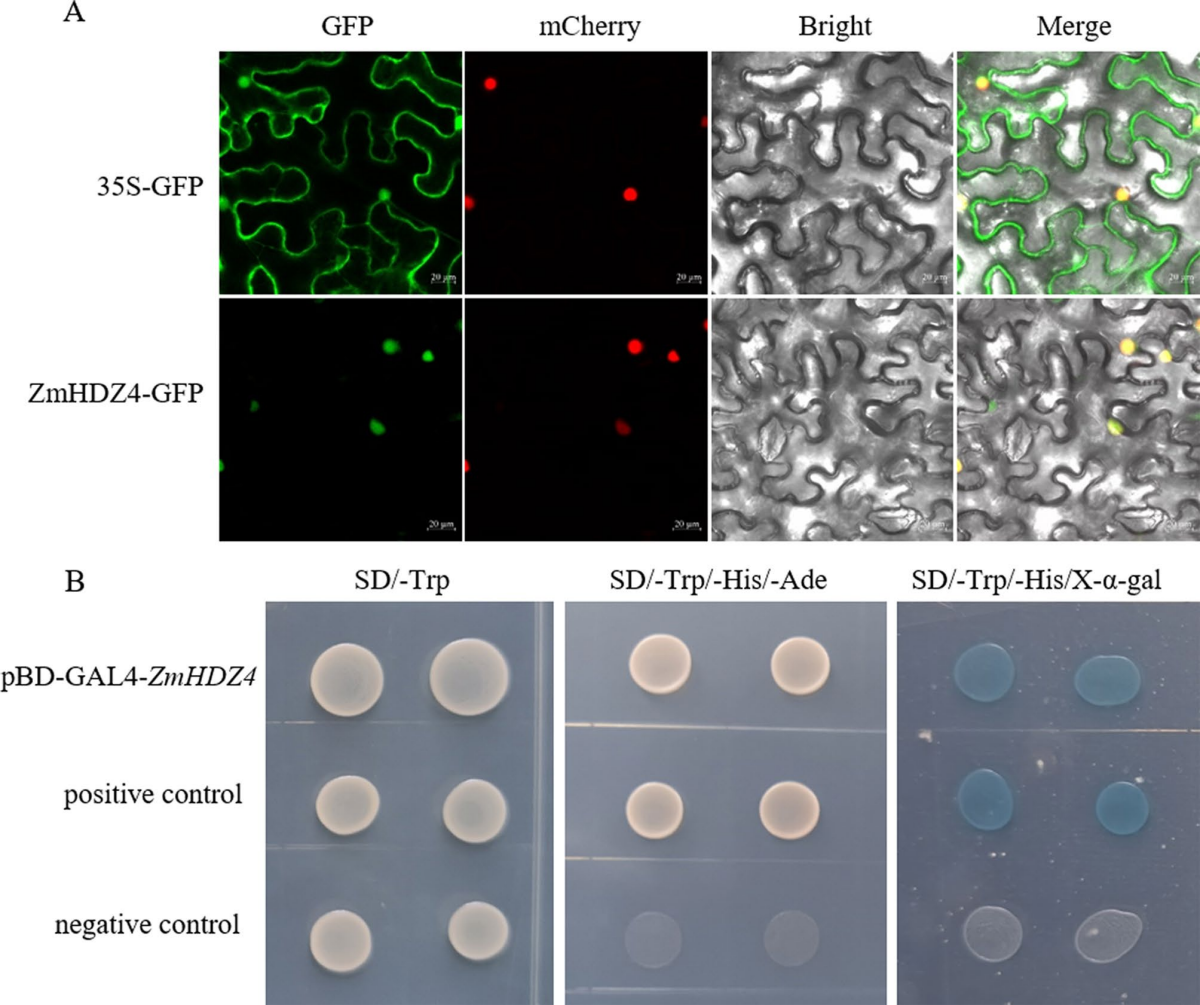
Subcellular localization and transcriptional activity analysis of ZmHDZ4. (**A**) Subcellular localization of ZmHDZ4. Scale bar represents 20 μm. (**B**) Transcriptional activity analysis of ZmHDZ4 protein by using yeast system. Negative control: pGBKT7 (empty vector). Positive control: pGAL4 (a full GAL4 transcription factor, encompassing both the transcriptional activation domain and the DNA-binding domain)

The pMDC83-*ZmHDZ4*-GFP vector was constructed to investigate the subcellular localization of ZmHDZ4, and the result showed that the *ZmHDZ4*-GFP fusion protein exhibited fluorescence signals exclusively in the nucleus (Fig. 2A). To identify the transcriptional activation role of *ZmHDZ4*, the constructed pBD-GLA4-*ZmHDZ4* vector was transformed into the yeast strain AH109. The transformants with the pBD-GLA4-*ZmHDZ4* and pGAL4 (positive control) grew well on SD/-Trp, SD/-Trp/-His/-Ade, and SD/-Trp/-His/-Ade/X-gal, and colonies on SD/-Trp/-His/-Ade/X-gal exhibited galactosidase activity. In contrast, the yeast cells carrying negative control (pGBKT7 empty vector) were only viable on the SD/-Trp medium. These findings conclusively demonstrate that ZmHDZ4 is a transcription factor with a transcriptional activation activity (Fig. 2B).

### Overexpression of ZmHDZ4 enhances drought tolerance in maize

**Fig. 3:**
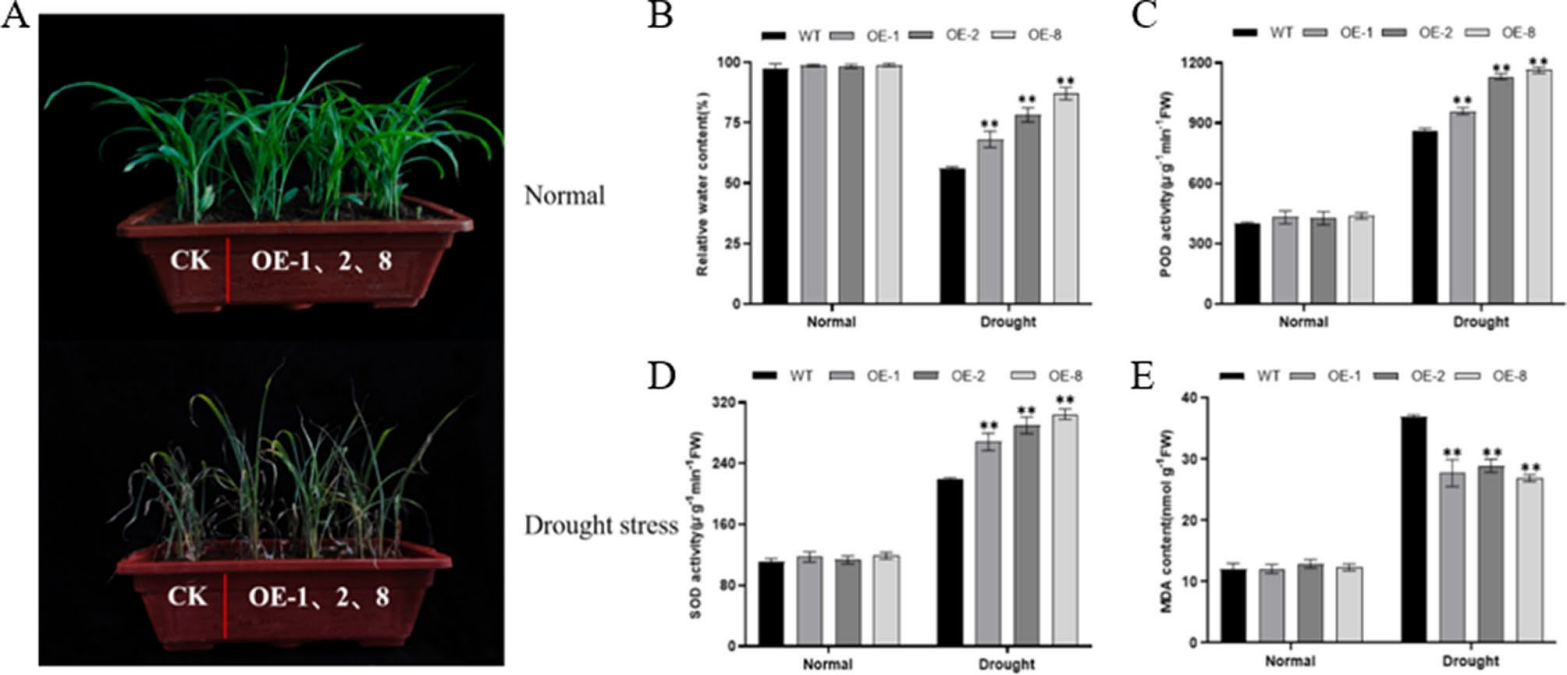
*ZmHDZ4* responses to drought stress. **A** Phenotypes of ZmHDZ4-OE and WT plants under drought and normal-watered conditions. **B** relative water content. **C** Leaf POD content. **D** SOD content in leaves. **E** MDA content

Transgenic maize lines overexpressing *ZmHDZ4* (*ZmHDZ4-*OE) under the control of the *CAMV35S* constitutive promoter were developed for functional identification. Due to the differences in the specific integration sites of the inserted genomic DNA and the T-DNA copy number, the relative expression level of *ZmHDZ4* in each line varied (Fig. S1A, B). Three transgenic lines namely OE-1, OE-2, and OE-8 were selected and subjected to drought stress treatment. At the three-leaf stage, both the *ZmHDZ4*-OE and wide-type seedlings were divided into two groups. The first group of plants received regular irrigation, while the second group of plants was exposed to drought stress by withholding irrigation for 15 days. There were significant phenotypic differences between the *ZmHDZ4*-OE and WT plants after 15 days of drought stress induction. Specifically, the leaves of *ZmHDZ4*-OE plants displayed less wilted, milder yellowing, and remained upright compared to the WT plants. Conversely, there were no phenotypic differences in growth status within each line in the well-watered treatment (Fig. 3A).

To further verify the role of *ZmHDZ4* under drought stress, the drought-related physiological parameters were measured. Under well-watered conditions, there were no significant differences in RWC between the *ZmHDZ4*-OE and WT plants; however, under drought-stressed conditions, the RWC of the *ZmHDZ4*-OE plants was significantly higher than that of the WT plants, with an increase of 20.23%, 39.54%, and 55.38% noted in the OE-1, OE-2, and OE-8 transgenic lines, respectively (Fig. 3B). The activities of POD and SOD of *ZmHDZ4*-OE plants were also significantly promoted compared to those of the WT plants (Fig. 3C, D). Malondialdehyde (MDA) is a harmful substance that accumulates in plants under drought-stressed conditions that can destroy the cell membranes stability. Under the well-watered conditions, there was no significant difference in the MDA content between the *ZmHDZ4*-OE lines and the WT plants. However, under drought-stressed conditions, the MDA content was significantly increased in both the *ZmHDZ4*-OE lines and the WT plants Notably, the increase was significantly lower in the *ZmHDZ4*-OE lines (33.12%, 27.64%, and 37.12% in the OE-1, OE-2, and OE-8 transgenic lines, respectively) compared to the WT plants (Fig. 3E). These results indicate that the overexpression of *ZmHDZ4* can increasing the activity of POD and SOD, thereby mitigating MDA production and providing a basic cellular protection mechanism, ultimately enhancing maize’s tolerance to drought stress.

### Identification of ZmHDZ4 binding motifs by DAPseq analysis

**Fig. 4:**
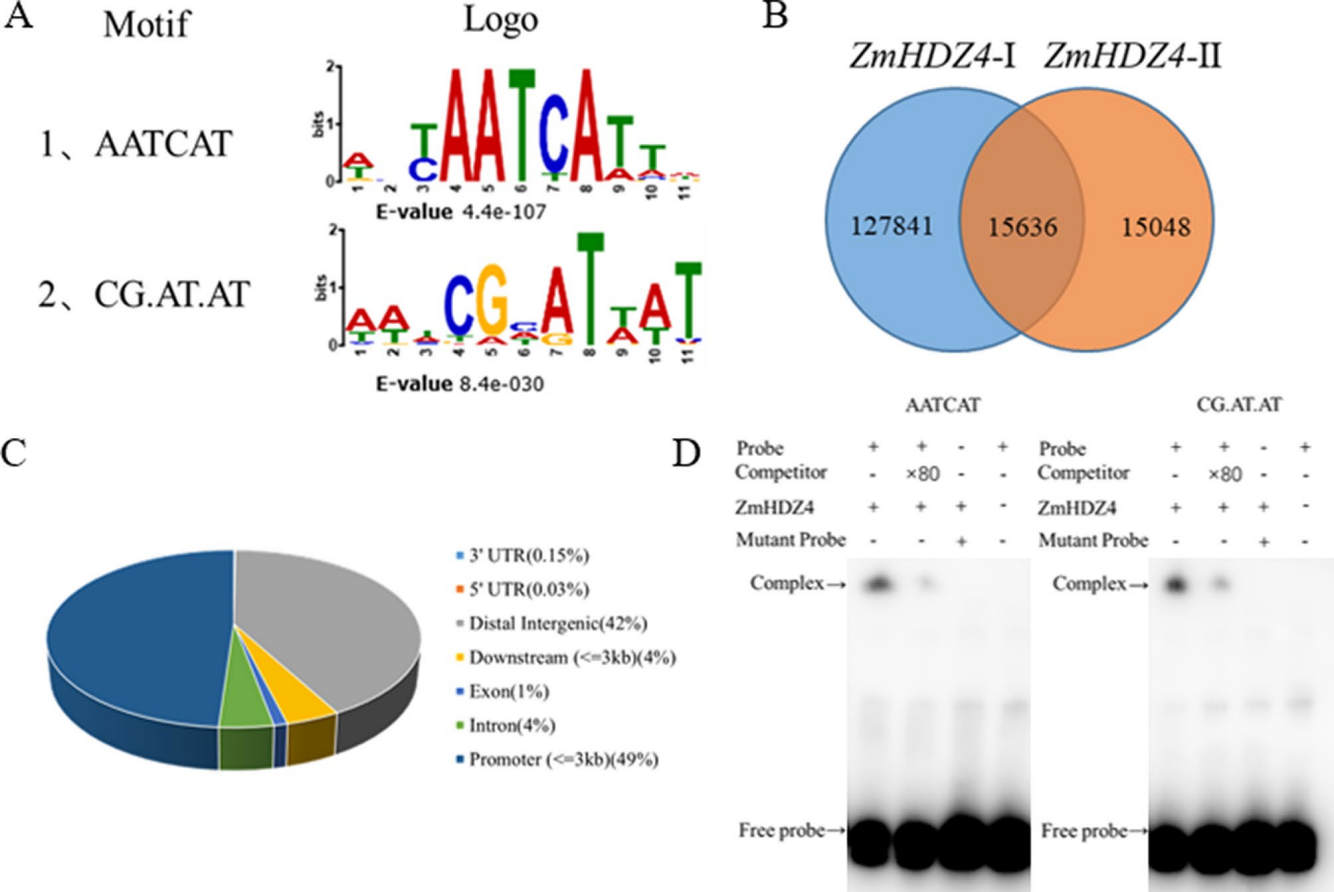
DAP-seq and EMSA analysis of ZmHDZ4. **A** ZmHDZ4 genome-wide binding motif in maize. **B** ZmHDZ4 genome-wide binding sites in maize. **C** Location of genome-wide binding sites in maize. **D** Electrophoretic mobility shift assay of ZmHDZ4 protein

To reveal the downstream target genes interacting with *ZmHDZ4*. DAP-seq was conducted. A total of 32.08 and 33.96 million reads were obtained from two biological replicates. Of these reads, 31.92 million and 33.79 million unique reads were aligned to the maize genome, yielding alignment rates of 99.39% and 99.37%, respectively (Table S2). A total of 15,636 peaks were identified across the 10 maize chromosomes (Fig. 4A, Table S3). Notably, 49% of these peaks were located in the promoter regions, while 0.18% were in untranslated regions (UTRs), 4% were downstream of the transcription termination sites, 4% were in the introns, 1% was the exons, and 42% were in distal gene gaps (Fig. 4B). Using the MACS2 software, two candidate binding sites —AATCAT and CG.AT.AT —were identified and validated by EMSAs (Fig. 4C, D). The result showed that *ZmHDZ4* could specifically bind to the motifs of AATCAT and CG.AT.AT.

### Validation of ZmHDZ4 downstream target genes

**Fig. 5:**
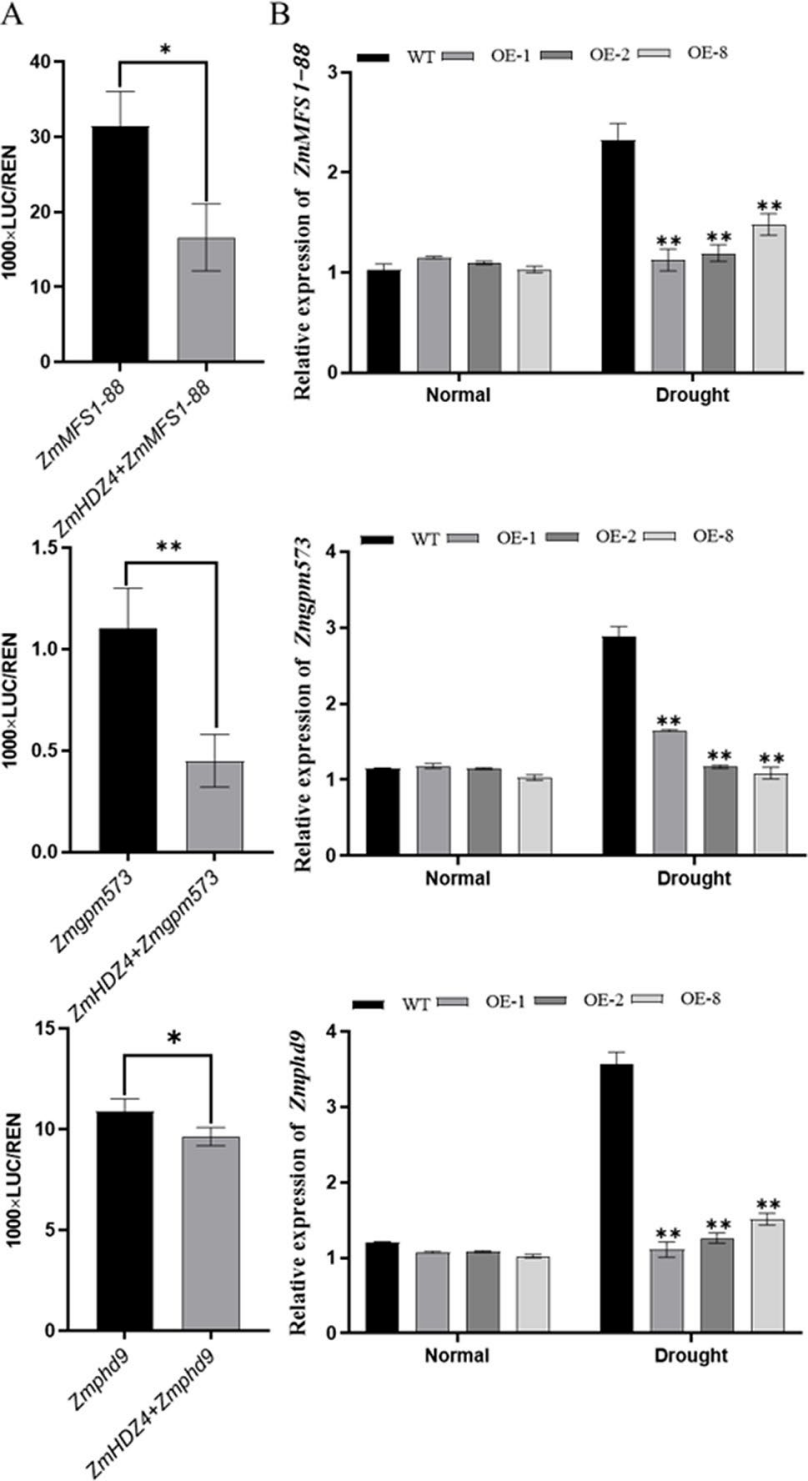
Analysis of the regulation of promoter activity of target genes by *ZmHDZ4*. **A** Dual luciferase system reporter assay. **B** qRT-PCR test; (**, P < 0.01; *, P < 0.05)

Based on the binding motifs identified by DAP-seq and EMSAs, coupled with gene functional annotation, three potential drought-related target genes were identified: Zm00001d020277 (*ZmMFS1-88*), Zm00001d004196 (*ZmGPM573*), and Zm00001d026270 (*ZmPHD9*), which all their promotor region contained the binding motif of AATCAT. To further explore the regulatory role of *ZmHDZ4* on these target genes, a dual luciferase assay was conducted. The vectors (promoter-0800 and *ZmHDZ4*-GFP) were constructed and separately transformed into the GV3101 *Agrobacterium tumefaciens* strain in a state of competence. After adjusting the bacterial liquid activity, the liquid was injected into young and healthy tobacco leaves. After approximately 48 h of normal cultivation, protein extraction was performed on the injected part of the leaves, and the relative ratio of firefly luciferase activity to Renilla luciferase activity was measured using a fluorescence spectrometer. The results showed that *ZmHDZ4* inhibited the promoter activity of *ZmMFS1-88*, *ZmGPM573*, and *ZmPHD9* (Fig. 5A). To further elucidate the regulatory relationship, the expression level of *ZmHDZ4*, *ZmMFS1-88*, *ZmGPM573*, and *ZmPHD9* were analyzed in the *ZmHDZ4*-OE and WT plants under both well-watered and drought-stressed condition using RT-qPCR assay. The results showed that under drought-stressed conditions, the expression levels of *ZmMFS1-88*, *ZmGPM573*, and *ZmPHD9* were significantly repressed in the *ZmHDZ4*-OE plants compared to those in the WT plants. However, there were no significant differences in the expression levels of these genes under the well-watered conditions (Fig. 5B), indicating that *ZmHDZ4* suppresses the expression of *ZmMFS1-88*, *ZmGPM573*, and *ZmPHD9*.

## Discussion

### ZmHDZ4 is involved in the drought stress tolerance regulation in maize

Drought is one of the most important abiotic stresses that severely affects crop growth and productivity. Shan Lun pioneered the concept of biological water conservation, which entails that the compensatory or super-compensation effect of the living organism itself can be used to achieve water conservation under drought stress, while the core concept of the viewpoint is that the living organism itself must be drought resistant, so the fundamental way to improve the water use efficiency of crop plants is to harness their intrinsic drought tolerance, physiological attributes, and gene potential to combat external water stress. Therefore, screening and identifying drought-tolerance genes is the basis for enhancing drought stress tolerance.

The *HD-ZipI* gene family plays a key role in regulating plant development, signal networks, and responses to various abiotic stresses triggered by endogenous and external stimuli [31, 37]. Under drought-rewatering treatment, Qiu et al., conducted a systematic analysis of the maize *HD-ZIP* gene family and found that *ZmHDZ4*, -6, -9, -14, -27, -32 and − 40 were key regulatory genes under drought stress [20]. To further explore the function of *ZmHDZ4*, *ZmHDZ4*-overexpressing transgenic lines were developed through genetic transformation in this study. Phenotype observation and related physiological and biochemical indices analysis were performed between *ZmHDZ4*-OE and wide-type plants under well-watered and drought-stressed conditions. RWC is considered a potential indicator of plant water status, and its decline can lead to limited cell proliferation, resulting in the reduction of plant growth [18, 22]. In this study, the growth of the wide-type plants was significantly inhibited under drought-stressed conditions and the degree of leaf damage was significantly more severe than that of the *ZmHDZ4*-OE plants. Additionally, the RWC of the wide-type plants was significantly lower than that of the *ZmHDZ4*-OE plants. Furthermore, *ZmHDZ4*-OE plants accumulated less MDA content after exposure to drought-stressed conditions compared to wide-type plants, potentially due to better maintenance of cell membrane integrity in the *ZmHDZ4*-OE plants under drought-stressed conditions. Reactive oxygen species (ROS) serve as effective signalling molecules that regulate plant metabolic processes. However, stringent control over ROS formation and scavenging is essential due to their enhanced reactivity and toxicity at high concentrations [1]. Plants combat excessive ROS and maintain internal balance through their antioxidant defence systems when facing abiotic stresses. SOD serves as the frontline enzyme against ROS. When plants are exposed to drought stress, SOD can detoxify the hydroxyl radical into H_2_O_2_, which is subsequently degraded by catalase (CAT) and POD into water and oxygen [18]. In this study, the activity of SOD and POD in the *ZmHDZ4*-OE plants was significantly higher than that in the wide-type plants under drought-stressed conditions, indicating that the overexpression of *ZmHDZ4* may specifically regulate the activity of some active oxygen clearance enzymes, thereby clearing excess ROS. In summary, *ZmHDZ4* is involved in the regulation to drought tolerance in maize.

### ZmHDZ4 enhances drought tolerance in maize by participating in osmotic regulation, sugar metabolism pathways, and hormone regulation

Transcription factors (TFs) have become the focus of research in recent years. By interacting with cis-acting elements within the promoters of target genes, TFs play an important role in plant stress response by regulating the expression of their target genes, up or down-regulated. Advances in high-throughput sequencing, proteomics and enhanced efficiency of genetic transformation techniques also offer robust technical support for elucidating gene functions and regulatory mechanisms.

In this study, the overexpression of *ZmHDZ4* was found to enhance drought tolerance in maize. Utilizing DAP-seq and EMSA, three target genes of *ZmHDZ4* were identified, each containing binding site motifs (Fig. 4D, Fig. S2). The dual-luciferase assay and RT-qPCR revealed that *ZmHDZ4* inhibits the expression of *Zm00001d020277* (*ZmMFS1-88*), *Zm00001d004196* (*ZmGPM573*) and *Zm00001d026270* (*ZmPHD9*). *ZmMFS1-88* belongs to the major facilitator superfamily (MFS), one of the largest superfamilies of membrane transporters to date; however, only a limited number of plant MFS transport proteins have been identified, primarily involved in the transport of sugars, oligopeptides, and nitrates. Sugars serve as signaling molecules that regulate plant growth and development, and evidence indicates that sugar transporters play pivotal roles in plant responses to various abiotic and biotic stresses [5, 40]. Remy et al. [23] elucidated the crucial role of *ZIFL1*, an *Arabidopsis* MFS member, in modulating both auxin polar transport and drought stress tolerance through alternative splicing of ZIFL1 [23]. Hou et al. [10] demonstrated that ZmMFS_1–62 and ZmMFS_1–73, proton-coupled folate transporters (PCFT), disrupted folate homeostasis and reduced tolerance to drought and salt stresses in maize seedlings, indicating that ZmMFS_1–62 and ZmMFS_1–73 are essential for drought and salt stress tolerance [10]. These findings suggest that *ZmMFS_1–88* may play a role in the regulation of drought stress responses.

Drought can induce systemic changes in metabolic networks, encompassing transamination, the TCA cycle, gluconeogenesis/glycolysis, glutamate-mediated proline biosynthesis, among others. The glycolysis/gluconeogenesis pathway is particularly crucial in regulating carbohydrate metabolism under drought stress [41, 42]. GPM573 is a member of the FMN-dependent oxidoreductase superfamily protein, which features a TIM-like beta/alpha barrel domain, a major structural family akin to phosphoglycerate isomerase (TPI). Phosphoglycerate isomerase, characterized by eight alternating β-sheets and α-helices forming a TIM barrel, plays a crucial role in glycolysis by interconverting glyceraldehyde 3-phosphate (G3P) and dihydroxyacetone phosphate (DHAP) [3, 26]. Li et al. [12] examined the transcriptomic and metabolic profiles of two inbred lines (si287, drought-tolerant, X178, drought-sensitive) subjected to drought stress during the 3-leaf stage. Their KEGG pathway analysis of genes and metabolites revealed significant differences in glycolysis/gluconeogenesis, flavonoid biosynthesis, starch and sucrose metabolism, and amino acids biosynthesis [12]. Studies have shown that drought stress modifies the abundance of glycolytic enzymes, including triose phosphate isomerase and phosphoglycerate kinase [43]. The expression of *ZmFBP* and *ZmPGM2*, both integral to glycolysis/gluconeogenesis, was significantly downregulated under drought stress conditions [12].

Zinc-finger proteins are prevalent in eukaryotes and play a crucial role in plant growth, development, and abiotic stress response. Classified according to conserved cysteine and histidine residues, Zinc-finger proteins encompass several types, including RING, LIM and PHD [35]. PHD zinc finger protein *VIL1* negatively regulates the ABA response, and the *vil1 Arabidopsis* mutants exhibit hypersensitivity to ABA during seed germination and enhanced drought tolerance [38]. In transgenic *Arabidopsis*, six soybean PHD proteins have been identified as regulatory factors in ABA signalling, conferring salt tolerance [30]. Ectopic expression of *MtPHD6* in *Arabidopsis* enhances tolerance to osmotic and drought stresses [21]. ZmPHD22, -30, and − 39 were strongly up-regulated under drought stress treatment [44]. StPHD59 showed significant down regulation in response to drought stress [45]. By suppressing the expression of *ZmPHD9*, *ZmHDZ4* appears to modulate the ABA signalling pathway, thereby improving drought tolerance.

Although experiments suggest that ZmHDZ4 possesses transcriptional activation activity, we noted instances of transcriptional repression by ZmHDZ4 on three target genes. Actually, this phenomenon is indeed observable in experiments. In Wang’s study [46], experiments conducted in yeast demonstrated that ZmERF21 exhibits transcriptional activation activity. However, ZmERF21 also exerts inhibitory regulation on its target genes. Analysis revealed the presence of a known EAR element within the ZmERF21 protein sequence. The EAR element is a well-established structural domain with transcriptional repressive activity, which explains its ability to both promote and repress target genes. Given this, we performed a protein sequence analysis on ZmHDZ4. However, we did not find the known EAR element. We hypothesize that ZmHDZ4 might harbor an undiscovered transcriptional repressive element, conferring upon it an inhibitory function. This hypothesis requires further experimental validation.

## Conclusions

In conclusion, *ZmHDZ4* positive regulates the drought tolerance in maize. The activity of SOD and POD in the *ZmHDZ4*-OE plants was significantly higher than that in the WT plants under drought-stressed conditions. Three candidate target genes of *ZmHDZ4* were firstly identified, indicating that the overexpression of *ZmHDZ4* may participate in osmotic regulation, sugar metabolism pathways, and hormone regulation.

## Electronic supplementary material

Below is the link to the electronic supplementary material.

Supplementary Material 1

Supplementary Material 2

## Data Availability

The DAP-Seq raw data of ZmHDZ4 have been deposited in the NCBI Sequence Read Archive (PRJNA1158686)(http://www.ncbi.nlm.nih.gov/bioproject/1158686).

## Abbreviations

DAP-Seq: DNA affinity purification sequencing
EMSA: The electrophoretic mobility shift assay
Dual-Luc: Dual-luciferase transient transcriptional activity assay
PHD: Plant homeodomain
ROS: Reactive oxygen species
POD: Peroxidase
SOD: Superoxidase dismutase
CAT: Catalase
MDA: Malondialdehyde (MAD)
