# Supplementary figures and images for: Functional study of ZmHDZ4 in maize (Zea mays) seedlings under drought stress
Source: BMC Plant Biol. 2024 Dec 19;24:1209. doi: 10.1186/s12870-024-05951-3 (PMC11656746; doi:10.1186/s12870-024-05951-3)

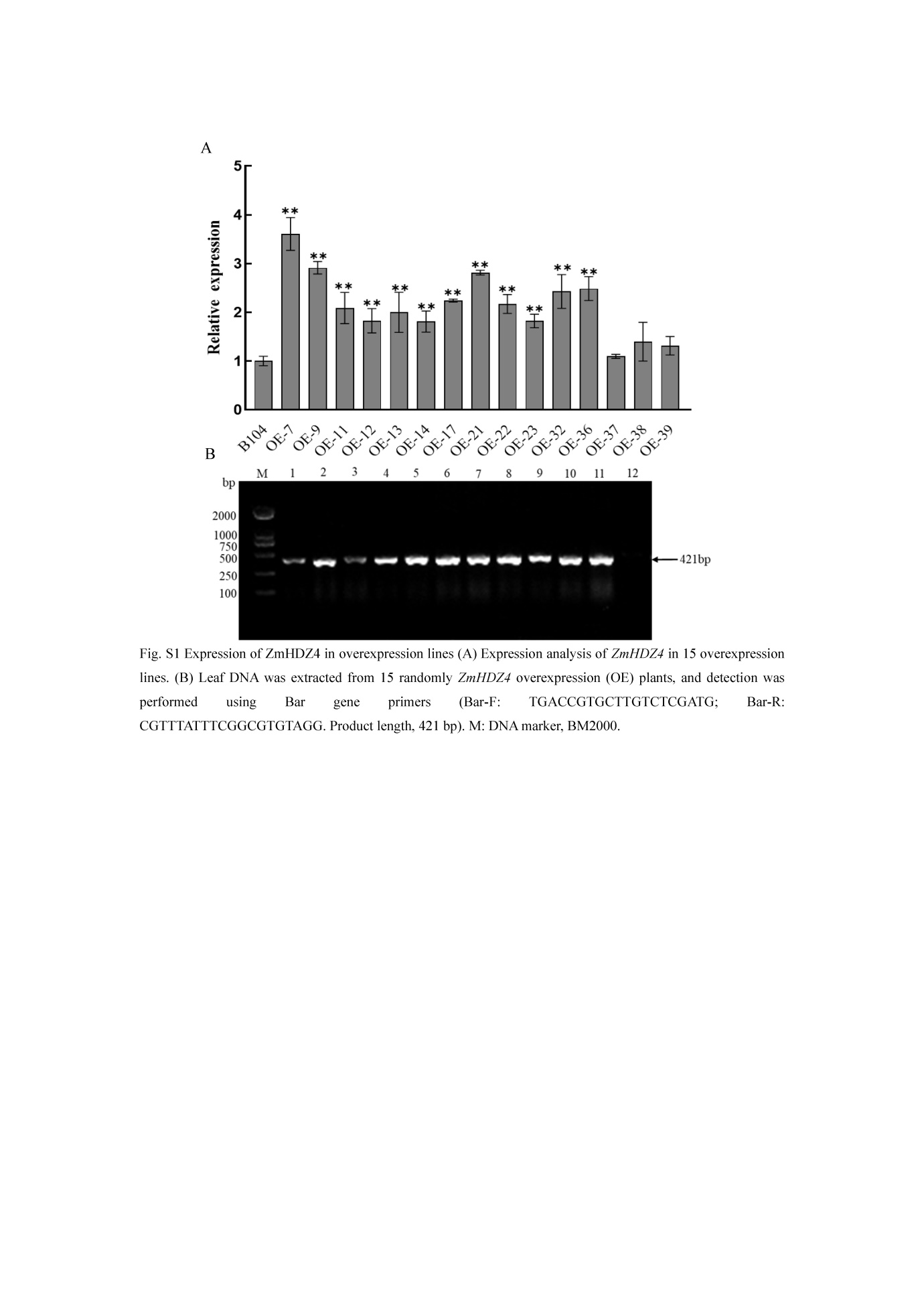


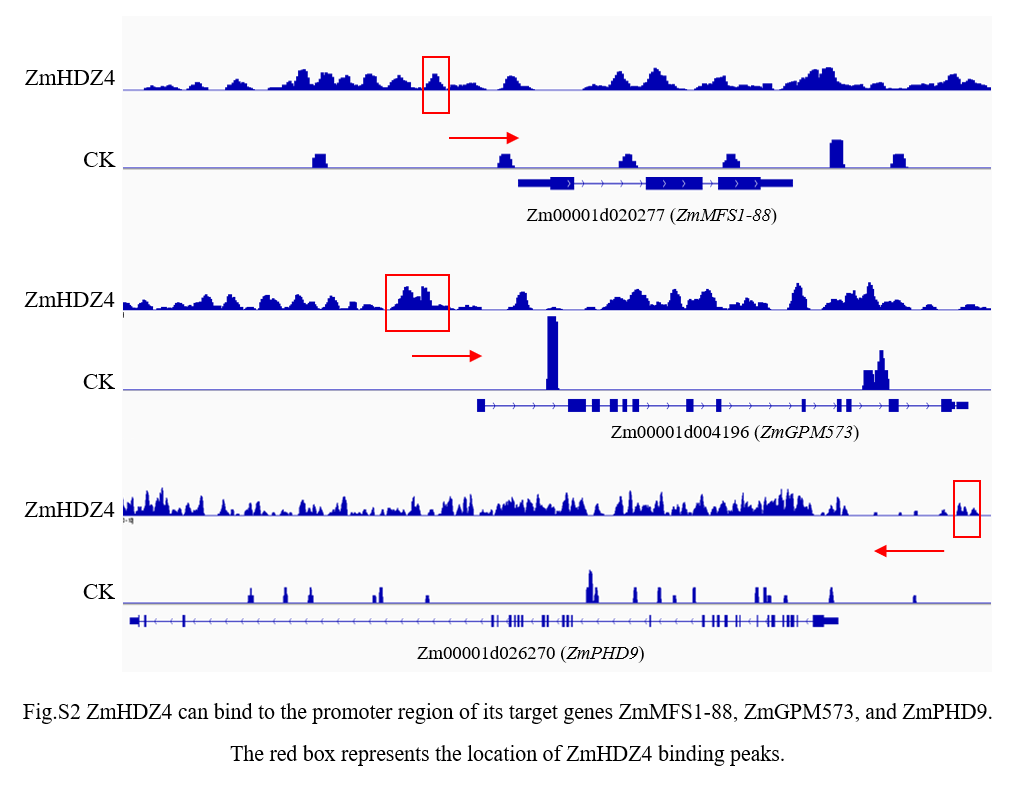

Supplement: Supplementary file 2 — Supplementary Material 2 [file 12870_2024_5951_MOESM2_ESM.docx]
